# Como Podemos Modular Favoravelmente o Ácido Úrico Sérico?

**DOI:** 10.36660/abc.20210739

**Published:** 2021-10-06

**Authors:** Luiza Antoniazzi, Laís Ferreira Dias

**Affiliations:** 1 Hospital das Clínicas Faculdade de Medicina Universidade de São Paulo São PauloSP Brasil Instituto do Coração do Hospital das Clínicas da Faculdade de Medicina da Universidade de São Paulo, São Paulo, SP – Brasil

**Keywords:** Acido Úrico/uso terapêutico, Dieta, Pessoa de Meia Idade, Fatores de Risco, Hipertensão, Hereditariedade, Hiperuricemia, Prevenção e Controle

O estudo “Impacto da Concentração de Ácido Úrico Sérico no Risco de Doença Cardiovascular: Um Estudo Coorte Realizado no Norte da China”,^1^ realizado em uma coorte de chineses de meia-idade, discute a influência do ácido úrico nos desfechos cardiovasculares. O estudo mostrou que o ácido úrico sérico foi um fator de risco para doenças cardiovasculares em pessoas de meia-idade e idosos no norte da China.^1^ Os achados sobre essa associação são controversos. Tendo sido comprovado em estudos anteriores.^2 - 5^

Desde 2018, a Diretriz Europeia para o controle da hipertensão recomenda o nível sérico de ácido úrico como um parâmetro para avaliar o risco cardiovascular,^6^ e a dosagem do ácido úrico deve ser incluída no acompanhamento de rotina de pacientes com hipertensão.

Deve-se considerar que fatores como atividade física, dieta, estresse e histórico familiar influenciam os níveis séricos de ácido úrico e também o risco cardiovascular; entretanto, eles não foram avaliados no estudo de Nie et al.,^1^ Dentre esses fatores, a dieta tem uma influência importante e já foi investigada em estudos anteriores.

A dieta *Dietary Approach to Stop Hypertension* (DASH), originalmente indicada para reduzir a pressão arterial, caracterizada pelo alto consumo de vegetais, grãos integrais e laticínios com baixo teor de gordura, também pode ser sugerida para reduzir os níveis de ácido úrico, como descrito por Gao et al.,^7^ com 71.893 participantes chineses no estudo Kailuan I e no estudo Kailuan II, que não apresentavam gota em uma avaliação anterior. A adesão à dieta DASH foi associada de maneira independente a uma baixa probabilidade de apresentar hiperuricemia. Outro estudo com adultos pré-hipertensos ou hipertensos mostrou uma redução nos níveis de ácido úrico gravemente elevados após 30 dias de adesão à dieta DASH, e esse efeito foi mantido por mais 90 dias.^8^

Quanto ao padrão alimentar vegetariano, um estudo com 424 individuos que consumiam carne, 425 que consumiam peixe, 422 vegetarianos e 422 veganos, participantes da coorte EPIC-Oxford, sugeriu que os veganos tinham os níveis séricos mais elevados de ácido úrico, seguidos por aqueles que consumiam carne, os que consumiam peixe e vegetarianos.^9^ Esses resultados contrastam com as descobertas de um estudo com 14.809 indivíduos (6.932 homens e 7.877 mulheres) participantes da pesquisa *Third National Health and Nutrition Examination Survey* (1988-1994), onde um alto consumo de carne e frutos do mar foi associado a níveis mais elevados de ácido úrico no sangue, embora não tenha havido associação com a proteína total da dieta.^10^

Essa controvérsia entre os achados e o fato de que veganos e vegetarianos apresentaram os valores extremos de uricemia no estudo EPIC-Oxford^9^ pode ser devido ao fato de que o alto consumo de laticínios integrais, leite integral, laticínios com baixo teor de gordura, leite com baixo teor de gordura, iogurte com baixo teor de gordura e queijos estão associados a um risco menor de hiperuricemia.^11^

A dieta mediterrânea, amplamente estudada nas doenças cardiovasculares, contribui para a prevenção e o tratamento da hiperuricemia. Uma maior adesão à dieta foi relacionada à menor probabilidade de ter hiperuricemia em 2.380 participantes do estudo ATTICA sem fatores de risco cardiovascular anteriores.^12^

Além do padrão alimentar, outros fatores podem influenciar o aumento do ácido úrico sérico. No estudo *Elderly-SEPHAR III Study* , com uma amostra de 1.920 adultos, dos quais 447 eram pacientes idosos (> 65 anos de idade), a idade representou um dos fatores que contribuíram para o aumento do nível de ácido úrico sérico.^13^ Em outro estudo, o índice de massa corporal, a circunferência da cintura e a relação cintura-altura foram positivamente associados ao risco de hiperuricemia em 2.895 participantes do estudo *China Health and Retirement Longitudinal Study* com 4 anos de acompanhamento.^14^

As doenças cardiovasculares continuam sendo a principal causa de morte no mundo,^15^ e as mudanças dietéticas que estão ocorrendo e continuarão ocorrendo em todas as partes do mundo, com predomínio de padrões alimentares que por si só possuem um perfil nutricional relacionado ao desenvolvimento de doenças crônicas e também de uma composição que favoreça o aumento do ácido úrico sérico, agravará o risco cardiovascular associado. Diante desse contexto, os achados do estudo de Nie et al., 2021, devem ser observados e utilizados para subsidiar ações tanto no manejo clínico de pacientes com risco cardiovascular, quanto para direcionar ações de saúde pública voltadas para sua prevenção.
