# Supplementary material for: Como Podemos Modular Favoravelmente o Ácido Úrico Sérico?
Source: Arq Bras Cardiol. 2021 Oct 6;117(4):676–7. [Article in Portuguese] doi: 10.36660/abc.20210739 (PMC8528355; doi:10.36660/abc.20210739)
Supplement: Translation

# Translation: How Can We Favorably Modulate Serum Uric Acid?

**Authors:** Luiza Antoniazzi, Laís Ferreira Dias

**Affiliations:** 1 Brazil Instituto do Coração do Hospital das Clínicas da Faculdade de Medicina da Universidade de São Paulo, São Paulo, SP – Brazil

**Keywords:** Uric Acid/therapeutic use, Diet, Middle Aged, Risk Factors, Hypertension, Heredity, Hyperruricemia, Prevention and Control

The study “Impact of Serum Uric Acid Concentration on the Risk of Cardiovascular Disease: A Cohort Study Conducted in Northern China”,^1^ carried out with a cohort of middle-aged Chinese, discusses the influence of uric acid on cardiovascular outcomes. The study showed that serum uric acid was a risk factor for cardiovascular disease in middle-aged and elderly people in northern China.^1^

Findings about this association are controversial. It has been proven in previous studies.^2 - 5^

Since 2018, the European Guideline for the management of hypertension recommends serum uric acid level as a parameter for assessing cardiovascular risk,^6^ and uric acid measurement should be included in the routine follow-up of patients with hypertension.

It should be considered that factors such as physical activity, diet, stress and family history influence serum uric acid levels and also cardiovascular risk; however, they were not evaluated in the study by Nie et al.^1^ Among these factors, diet has an important influence and has been investigated in previous studies.

The Dietary Approaches to Stop Hypertension (DASH) diet, originally indicated for reducing blood pressure, characterized by high consumption of vegetables, whole grains and low-fat dairy, can also be suggested for reducing uric acid levels, as described by Gao et al.,^7^ with 71,893 Chinese participants in the Kailuan I study and the Kailuan II study, who did not have gout in a previous evaluation. Adherence to the DASH diet was independently associated with a low probability of having hyperuricemia. Another study with pre-hypertensive or hypertensive adults showed a reduction in severely high uric acid levels after 30 days of adherence to the DASH diet, and this effect was sustained for another 90 days.^8^

As for the vegetarian eating pattern, a study with 424 meat eaters, 425 fish eaters, 422 vegetarians and 422 vegans, participants of the EPIC-Oxford cohort, suggested that vegans had the highest serum levels of uric acid, followed by meat eaters, fish eaters and vegetarians.^9^ These results contrast with findings from a study of 14,809 participants (6,932 men and 7,877 women) from the Third National Health and Nutrition Examination Survey (1988–1994), where a high consumption of meat and seafood was associated with higher blood uric acid levels , although there was no association with total dietary protein.^10^

This controversy between the findings and the fact that vegans and vegetarians had the extremes of uricemia values in the EPIC-Oxford study,^9^ 2013), may be due to the fact that a high consumption of total dairy products, total milk, low-fat dairy products, low-fat milk, low-fat yogurt, and cheese is associated with a lower risk of hyperuricemia.^11^

The Mediterranean diet, widely studied in cardiovascular diseases, contributes to the prevention and treatment of hyperuricemia. Greater adherence to the diet was related to a lower probability of having hyperuricemia in 2,380 participants in the ATTICA study without previous cardiovascular risk factors.^12^

In addition to the dietary pattern, other factors can influence the increase in serum uric acid. In the Elderly-SEPHAR III Study, with a sample of 1,920 adults, of whom 447 were elderly patients (>65 years of age), age represented one of the factors contributing to the increased level of serum uric acid.^13^ In another study, body mass index, waist circumference, and waist-to-height ratio were positively associated with the risk of hyperuricemia in 2,895 participants in the China Health and Retirement Longitudinal Study with a 4-year follow-up.^14^

Cardiovascular diseases continue to be the leading cause of death in the world^15^ and dietary changes that are occurring and will continue to occur in all parts of the world, with a predominance of dietary patterns that in themselves have a nutritional profile related to development of chronic diseases and also a composition that favors an increase in serum uric acid, will aggravate the associated cardiovascular risk. Considering this context, the findings of the study by Nie et al., 2021, should be observed and used to support actions both in the clinical management of patients at cardiovascular risk, as well as to direct public health actions aimed at its prevention.
